# *Entamoeba histolytica *and *E. dispar *trophozoites in the liver of hamsters: in vivo binding of antibodies and complement

**DOI:** 10.1186/1756-3305-3-23

**Published:** 2010-03-26

**Authors:** Cássia AX Costa, Álvaro C Nunes, Anderson J Ferreira, Maria A Gomes, Marcelo V Caliari

**Affiliations:** 1 Programa de Pós-Graduação em Patologia, Instituto de Ciências Biológicas da Universidade Federal de Minas Gerais. Av. Antônio Carlos 6627, Belo Horizonte, Minas Gerais, Brasil; 2 Departamento de Patologia Geral, Instituto de Ciências Biológicas da Universidade Federal de Minas Gerais. Av. Antônio Carlos 6627, Belo Horizonte, Minas Gerais, Brasil; 3 Departamento de Biologia Geral, Instituto de Ciências Biológicas da Universidade Federal de Minas Gerais. Av. Antônio Carlos 6627, Belo Horizonte, Minas Gerais, Brasil; 4 Departamento de Morfologia, Instituto de Ciências Biológicas da Universidade Federal de Minas Gerais. Av. Antônio Carlos 6627, Belo Horizonte, Minas Gerais, Brasil; 5 Departamento de Parasitologia, Instituto de Ciências Biológicas da Universidade Federal de Minas Gerais. Av. Antônio Carlos 6627, Belo Horizonte, Minas Gerais, Brasil

## Abstract

**Background:**

Human amoebiasis is caused by the parasitic protozoan *Entamoeba histolytica *that lives in the large intestine of hosts, where can produce asymptomatic colonization until severe invasive infections with blood diarrhea and spreading to other organs. The amoebic abscesses in liver are the most frequent form of amoebiasis outside intestine and still there are doubts about the pathogenic mechanisms involved in their formation. In this study we evaluated the *in situ *binding of antibodies, C3 and C9 complement components on trophozoites, in livers of hamsters infected with *E. histolytica* or *E. dispar*. These parameters were correlated with the extension of the hepatic lesions observed in these animals and with trophozoites survivor.

**Methods:**

Hamsters were inoculated intra-hepatically with 100,000 trophozoites of *E. histolytica *or *E. dispar *strain and necropsied 12, 24, 48, 72, 144 and 192 h after inoculation. Antibodies, C3 and C9 binding to trophozoites were detected by immunohistochemistry. The estimation of the necrosis area and the number of labeled trophozoites was performed using digital morphometry analysis.

**Results:**

In the liver sections of animals inoculated with the amoebas, the binding of antibodies to *E. histolytica *trophozoites was significantly lower than to *E. dispar *trophozoites. Trophozoites of *E. dispar *were also more frequently vacuolated and high labeled cellular debris observed in the lesions. Positive diffuse reaction to C3 complement component was more intense in livers of animals inoculated with *E. histolytica *after 24 and 72 h of infection. C3^(+) ^and C9^(+) ^trophozoites were detected in the vascular lumen, granulomas and inside and in the border of necrotic areas of both infected group animals. C3^(+) ^and C9^(+) ^trophozoite debris immunostaining was higher in livers of *E. dispar *than in livers of *E. histolytica*. A positive correlation between necrotic areas and number of C9^(+) ^trophozoites was observed in animals inoculated with *E. dispar*.

**Conclusion:**

Morphological and immunohistochemical results suggest that antibodies and complement are able to bind and destroy some trophozoites in the liver of experimentally infected hamsters, perhaps selecting the more resistant parasites which are responsible by progression of amoebic abscesses. The findings indicate that *E. histolytica *possesses an enhanced ability *in vivo *to evade the immune responses compared to *E. dispar*, although it also causes experimental hepatic lesions.

## Background

Amoebiasis is caused by the protozoan parasite *Entamoeba histolytica *which resides in the host large intestine. The severity of this disease can range from an asymptomatic infection to invasive ulceration, colon inflammation and diarrhea associated with blood. The dissemination of trophozoites to the blood stream usually leads to the development of hepatic abscess which is the most frequent type of extra-intestinal amoebiasis [1]. The lesions observed during amoebiasis are caused by harmful products secreted by trophozoites and, possibly, by host defenses [2-5]. The mechanisms involved in generating lesions by *E. histolytica *are not still completely understood, as well as the role of immune responses raised against trophozoites.

In 1993, Diamond & Clark separated the *E. histolytica *species into two forms: the pathogenic *E. histolytica*, which is invasive and causes symptomatic disease and the non-pathogenic *E. dispar*, morphologically similar to the pathogenic one [6]. It has been shown that the *E. dispar *form might cause experimental lesions in livers [7-9].

The trophozoite survival and locomotion are influenced by its own secretion products as well as by host molecules such as extracellular matrix (ECM) proteins and immunologic molecules. The immunological system is very efficient in producing responses against microorganisms such as protozoan parasites. These responses involve antigen recognition and elaboration of a specific reaction aimed at eliminating such microorganisms. Activation of the innate immune response occurs through pathogen recognition receptors (PPRs) such as Toll-like receptors (TLR) that recognize and bind pathogen-associated molecular pattern (PAMP) domains of foreign microorganisms. This process initiates an inflammatory response [10] and seems to select resistant trophozoites, amplifying the hepatic lesions caused by amoeba infection [11].

At the moment, it is not known if the antibodies against trophozoites produced by patients with hepatic abscess can contribute to the selection of resistant trophozoites, leading to the destruction of the parenchyma at the beginning of the lesion development. These antibodies may persist within the circulation even after eradication of the amoebiasis and may not prevent a new infection [12].

The locomotion of trophozoites in the ECM is possibly due to the production and release of cysteine proteinases. These enzymes degrade many molecules such as collagen, elastine, fibrin and laminin [3] and interact with the immune system of the host through the cleavage of the C3 complement mediated by the neutral cysteine proteinase 56 kD [13] leading to production of molecules such as C3a and C5a that amplify the inflammatory process.

The antibodies and the complement system might not be able to suppress an *E. histolytica *infection, however they could destroy susceptible trophozoites. As a result, the primary function of the immune system is shifted, allowing the proliferation of the more resistant trophozoites and, consequently, the development of hepatic abscess. Thus, taking into account these considerations, the aim of this current study was to evaluate the *in situ *binding of antibodies, C3 and C9 complement components on trophozoites in livers of hamsters infected with *E. histolytica *or *E. dispar *and to correlate these parameters with the extension of the hepatic lesions and with trophozoites number observed in these animals in different time points. The use of *E. dispar *and comparative studies between the lesions caused by *E. histolytica *and *E. dispar *can amplify the knowledge about pathology in amoebiasis.

## Methods

### Maintenance, Growth and Inoculation of Trophozoites

Animals were obtained from the Instituto de Ciências Biológicas da Universidade Federal de Minas Gerais. Two groups of 30 hamsters 60-days old were inoculated via the intrahepatic route with 100,000 trophozoites of *E. dispar *MCR and *E. histolytica *EGG strains, respectively. Maintenance, growth and inoculation of trophozoites were made as described in previous work [7]. The *E. dispar *and *E. histolytica *strains were identified by zymodeme analysis and PCR [14,15]. All experimental protocols were performed in accordance with the guidelines for the humane use of laboratory animals from our Institute and approved by local authorities.

### Histopathological and Immunohistochemical Analysis

Twelve, 24, 48, 72, 144 and 192 hours after inoculation, five animals were sacrificed by cervical dislocation and the livers were removed. The left lobes of the livers were fixed in 10% buffered formaldehyde pH 7.2. After processing in alcohol and xylene, fragments were embedded in paraffin and 4-μm thick sections were obtained and processed for hematoxylin and eosin (H&E) and for immunohistochemistry.

In order to identify and quantify *E. histolytica *and *E. dispar *trophozoites in histological sections, the following immunohistological procedure was performed. Paraffin embedded liver sections from infected hamsters were deparaffinized, hydrated, and followed by a treatment with 3.5% PBS/H_2_O_2 _solution for blocking endogenous peroxidase during 20 min. Unspecific binding was blocked by goat serum diluted 1:40 for 30 min. *E. histolytica *and *E. dispar *trophozoites were identified in those sections by incubation with specific antisera raised in rat by inoculation of trophozoites from EGG and MCR strains, respectively, diluted 1:2,000, during 30 min at room temperature. The sections were incubated with biotinylated goat IgG anti-rat Igs diluted 1:50 during 30 min at room temperature (Zymed Laboratories Inc. San Francisco, Calif.), washed in PBS, pH 7.2 and incubated with streptavidin diluted 1:100 (Zymed Laboratories Inc.).

It is expected that *E. histolytica *or *E. dispar *trophozoites, present in the hepatic abscesses, are covered with antibodies produced by the infected host. To quantify the deposition of host antibodies on the parasite surface, the following procedure was performed. The sections were treated with rat anti-hamster antibodies, diluted to 1:50, during 30 min at room temperature. After washing with PBS, pH 7.2, the sections were treated with goat anti-rat antibodies conjugated with biotin diluted to 1:200 during 30 min at room temperature, washed once more in PBS and incubated with streptavidin diluted 1:100.

Activation and deposition of complement factors such as C3 or C9 on the parasites surface was also studied. The anti-C3 and anti-C9 antibodies were kindly provided by Dr J. Ding-E. Young - Rockefeller University, NY, USA. To investigate C3 deposition, the sections were treated with rabbit anti-C3, diluted 1:2,000, during 16-18 h at 4°C. After washing with PBS, sections were incubated with biotinylated anti-rabbit antibody (1:50, 4°C, Pharmingen, California, USA) for 30 min. Following PBS rinses, sections were incubated with streptavidin-horseradish peroxidase conjugate (Zymed Laboratories) for additional 30 min at 4°C. C9 factor deposition was investigated using anti-C9 antibody in a similar protocol as described above. In all reactions, the color was detected using a solution of 0.05% diaminobenzidine and 0.2% H_2_O_2 _at room temperature for 10 min.

Sections incubated without primary antibody were used as negative controls and sections obtained from infected livers with massive amount of trophozoites and from heart submitted to ischemia/reperfusion were utilized as positive controls for *E. histolytica*, *E. dispar*, C3 and C9, respectively. The linearity between the binding of primary antibodies and staining intensity was validated by using different concentrations of the primary antibody and times of enzymatic reaction of peroxidase/DAB, and direct correlations were observed.

### Myocardial Ischemia/Reperfusion

Under anesthesia with 10% ketamine and 2% xylazine (4:3, 0.1 ml/100 grams, i.p.), one rat was placed in the supine position on a surgical table, tracheotomized, intubated and ventilated with room air using a respirator for small rodents. The chest was opened by a left thoracotomy at the fourth or fifth intercostal space. To expose the heart, a small-sized retractor was used to maintain the ribs separated. After incision of the pericardium, the heart was quickly removed from the thoracic cavity and turned left to allow access to the proximal left anterior descending (LAD) coronary artery. A small clip was used to occlude the vessel for 15 min. After this period, the occlusion was released to allow the reperfusion of the heart for 5 min. Following the reperfusion, the heart was removed and fixed in 10% buffered formaldehyde pH 7.2 for posterior immunohistochemical analysis.

### Morphometry

The quantification of the necrosis area and of labeled trophozoites was performed using the digital morphometry analysis. To quantify the number of trophozoites, 30 frames of 53,333.4 μm^2 ^were randomly digitalized using a JVC-TK1270 microcamera and counted with the use of the KS300 software coupled to a Carl Zeiss image analyzer (Carl Zeiss, Oberkochen, Germany). The necrosis areas and trophozoites numbers were manually calculated using a digital pad. The C3 and C9 positive trophozoites counts were made with the same method.

To quantify the antibodies produced by the host covering the trophozoites in the hepatic abscess, we utilized the optical density (OD) calculation method. The following procedures were performed [16]:

• The RGB signal and the automatic gain control of the microcamera was deactivated to allow black and white capture through the KS300 software;

• A GG495 interference filter (Schott, Mainz, Germany) was used to measure the OD at the wavelength of 500 nm, which is absorbed only by the DAB reaction product.

• Images from sections lightly counterstained were captured with an ×40 objective;

• Only objects larger than 30 pixels were measured;

• The condenser aperture was calibrated during Kohler illumination, the geometric calibration was performed separately in the vertical and horizontal axes through a micrometric slide (Carl Zeiss), and the illumination levels were checked at 30 min intervals.

For each time point, 60 positive trophozoites were analyzed randomly. The algorism function was used to select the positive areas and creation of a binary image. OD was obtained using the ratio between the transmitted light and the incident light at 500 nm wavelength, according to the Beer's law:

Where I_0 _is the total intensity of light transilluminating the areas not marked of the specimen and I is the intensity of light transmitted from any given pixel of the analyzed area. In a digital specimen image, I is proportional to pixel grey value. Since each labeled trophozoites is composed of many pixels, the sum of grey value (SUMD) divided by the area (in pixels) gives us the I value. In fact, I will be equal to MEAND (densitometric mean). In the image analyzer utilized the brightness values range from 0 to 255 (8 bit range), thus the theoretical I_0 _is equal to 255. Consequently, I_0 _represents the mean pixel values of background tissue and it is obtained through the generation of a binary image from this region and subsequent calculation of MEAND. The background grey value of our material was equal to 141, thus the formula for OD calculation was:

Thus, OD values closer to zero correspond to strong immunolabeling and, consequently, more antibodies binding to trophozoites. OD values were expressed as grey units.

### Statistical Analysis

Data are expressed as mean ± SEM. Statistical significance for necrotic area and number of trophozoites data after 12 h of inoculation was estimated using Man-Whitney followed by the Least Significance Difference (LSD) test. The numbers of C3 and C9 positive trophozoites and binding of antibodies to trophozoites (OD) were analyzed utilizing the Kruskal-Wallis and Man-Whitney test followed by the LSD. Differences were considered significant at a p ≤ 0.05. The correlations among the parameters analyzed were evaluated by the Spearman test. Tests were performed with the SPSS 15.0 software package.

## Results

### Histopathology and Quantitative Analysis of the Hepatic Necrosis

**Figure 1:**
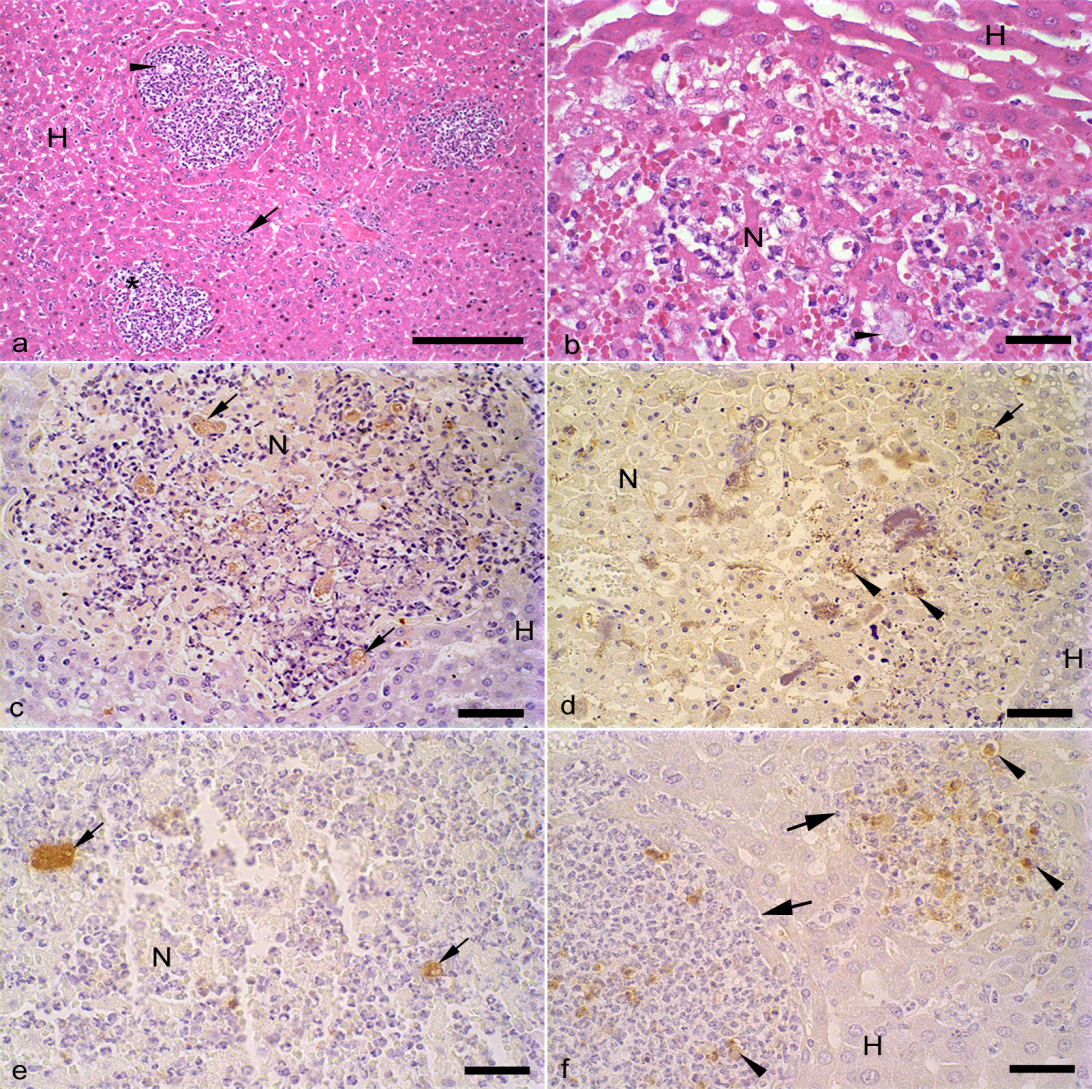
**Photomicrographs of liver sections from hamsters inoculated with *E. histolytica *or *E. dispar *trophozoites and sacrificed at 12 hs of infection**. (a) Circumscribed nodular lesions (*) contained inflammatory infiltrate constituted predominantly by neutrophils, cellular debris and scarce *E. histolytica *trophozoites (arrow head). It is possible to observe the presence of leucocytes inside of the sinusoidal capillaries (arrow). (H) non-necrotic parenchyma. H&E; Bar = 100 μm. (b) Necrotic area (N) produced by *E. dispar *with intense capillary congestion, inflammatory infiltrate and trophozoite (arrow head). (H) non-necrotic parenchyma with presence of dilated sinusoidal capillaries. H&E; Bar = 20 μm. (c) Immunohistochemical reaction against *E. histolytica *trophozoites showing nodular necrosis with small central area of necrosis (N) and trophozoites (arrows). (H) non-necrotic parenchyma. Bar = 50 μm. (d) Immunohistochemical reaction against *E. histolytica *trophozoites showing extensive area of hepatic necrosis (N). Trophozoite (arrow) and antigens derived from the trophozoites (arrow heads) are also observed. (H) non-necrotic parenchyma. Bar = 50 μm. (e) Immunohistochemical reaction against *E. dispar *trophozoites (arrows) showing central area of necrosis (N). Bar = 20 μm. (f) Immunohistochemical reaction against *E. dispar *trophozoites showing nodular lesions (arrows) delimited by non-necrotic hepatic parenchyma (H). Granular dark brown material indicates positive staining for *E. dispar *antigens (arrow heads). Bar = 20 μm.

At 12 h of infection, hepatic lesions were observed in all infected hamsters and their microscopic aspects were similar in both groups of animals. The lesions presented as delimited nodular necrosis areas constituted of cellular debris, trophozoites and discrete inflammatory infiltrate formed by neutrophils and macrophages. These nodular lesions were surrounded by normal hepatic parenchyma and were found mainly at 12 h after infection (Fig. 1a). The fusion of these focal lesions led to the formation of central areas of necrosis with a border composed by cellular debris, trophozoites and low amount of macrophages and neutrophils (Fig. 1b). Also, the inner parts of the central areas of necrosis showed liquefaction necrosis and/or coagulation and presence of trophozoites. The sinusoidal capillaries were dilated and presented a larger amount of leucocytes. In accordance, no significant differences in the extension of the necrotic area were observed in livers from hamsters inoculated with *E. histolytica* or *E. dispar *(*E. histolytica*: 5.2 × 10^5 ^± 7.2 × 10^5^; *E. dispar*: 3.1 × 10^6 ^± 2.7 × 10^6^; p > 0.05). The data corresponding to the number of trophozoites and extension of the necrotic areas after 24, 48, 72, 144, and 192 h of infection were collected and analyzed previously and they were used in the present study [7]. Neither macro- nor microscopic lesions were observed in animals inoculated with *E. histolytica *or *E. dispar *flora.

### Qualitative and Quantitative Immunohistochemical Analysis of Trophozoites

At 12 h of infection, trophozoites were found primarily in the border of the central areas of necrosis and, in small amount, inside of these areas, in nodular lesions and sinusoidal capillaries (Figs. 1c, d, and 1e). Positive immunoreactivity against *E. dispar *and *E. histolytica *antigens were observed in all regions described above (Figs. 1d and 1f), suggesting the presence of trophozoites debris (granular aggregates similar to cell membrane debris) or products of secretion of these parasites (shapeless zones).

The number of trophozoites was significantly higher in livers of *E. histolytica*- than *E. dispar*-infected hamsters at 12 h of infection (36.2 ± 19.9 *E. histolytica *trophozoites and 2.2 ± 2.3 *E. dispar *trophozoites; p < 0.05). Nevertheless, this difference was not observed in the other time points.

### Qualitative and Quantitative Analysis of in situ Binding of Antibodies to Trophozoites

**Figure 2:**
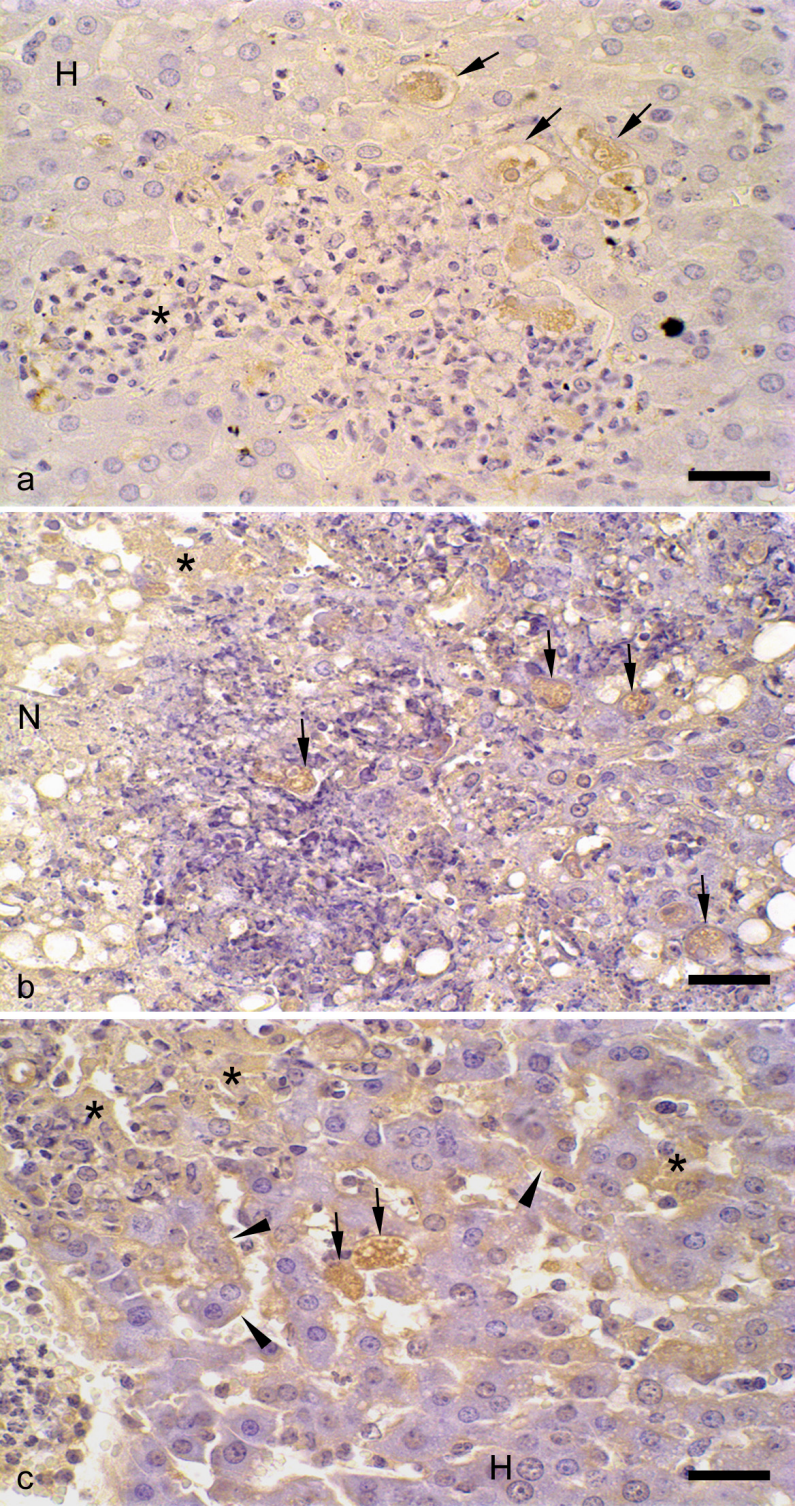
**Immunohistochemical reaction for antibodies bound to trophozoites in liver of hamsters infected with *E. histolytica *or *E. dispar *trophozoites**. (a) Nodular lesion constituted by neutrophils and macrophages (*) and circumscribed by non-necrotic hepatic parenchyma (H). Vacuolizated *E. dispar *trophozoites with positive immunoreactivity for bound antibodies without contact to inflammatory cells (arrow). (b) Necrosis area (N) containing positive *E. histolytica *trophozoites (arrows). Immunoreactivity was also observed in injured hepatocytes (*). (c) Hepatic parenchyma containing positive reaction for antibodies on sinusoidal capillaries walls (arrow heads), injured hepatocytes (*) and *E. histolytica *trophozoites (arrows). (H) non-necrotic parenchyma. Bar = 20 μm.

Immunohistochemistry technique revealed the presence of antibodies bound to trophozoites in all animals infected with *E. dispar *and *E. histolytica *with no apparent differences between both strains (Figs. 2a and 2b). *E. dispar *trophozoites frequently presented vacuoles, indicating degeneration of the parasites. Positive staining was also found in sinusoidal capillaries walls and in hepatocytes (Fig. 2c).

**Figure 3:**
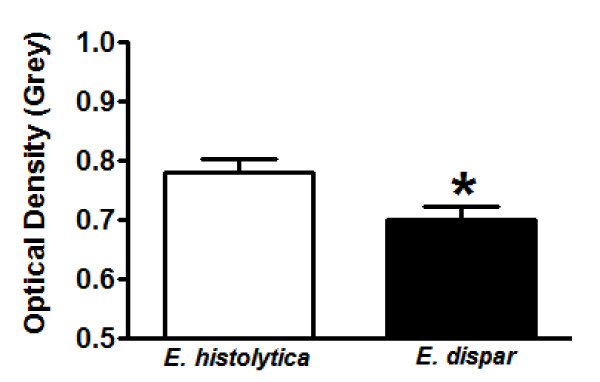
**Quantification of antibodies ligated on trophozoites in liver of hamsters infected with *E. histolytica *or *E. dispar *trophozoites**. (a) Average of the optical density (OD) values obtained at each time point (12, 24, 48, 72, 144, and 192 hs) of infection. OD values closer to zero correspond to strong immunolabeling and, consequently, higher anti-antibodies against trophozoites binding. *p < 0.05 vs *E. histolytica *strain. Data is shown as means ± SEM.

The antibody binding to trophozoites was significantly lower in animals inoculated with *E. histolytica *compared with *E. dispar *(*E. histolytica*: 0.77 ± 0.11 grey; *E. dispar*: 0.70 ± 0.11 grey; p < 0.05) (Fig. 3). Higher values of OD indicate lower densities of immunohistochemical labeling. As the OD scale is small, little differences among the values correspond to significant changes in the groups.

Correlation between bound antibodies and the number of trophozoites in lesions was verified by the Spearman rho coefficient. A positive correlation between OD and number of trophozoites was observed in hamsters inoculated with *E. histolytica*, i.e. large amount of trophozoites with low detection of binding of antibodies in trophozoites (p < 0.05).

### Qualitative and Quantitative Analysis of C3 and C9 Complement Deposition

**Figure 4:**
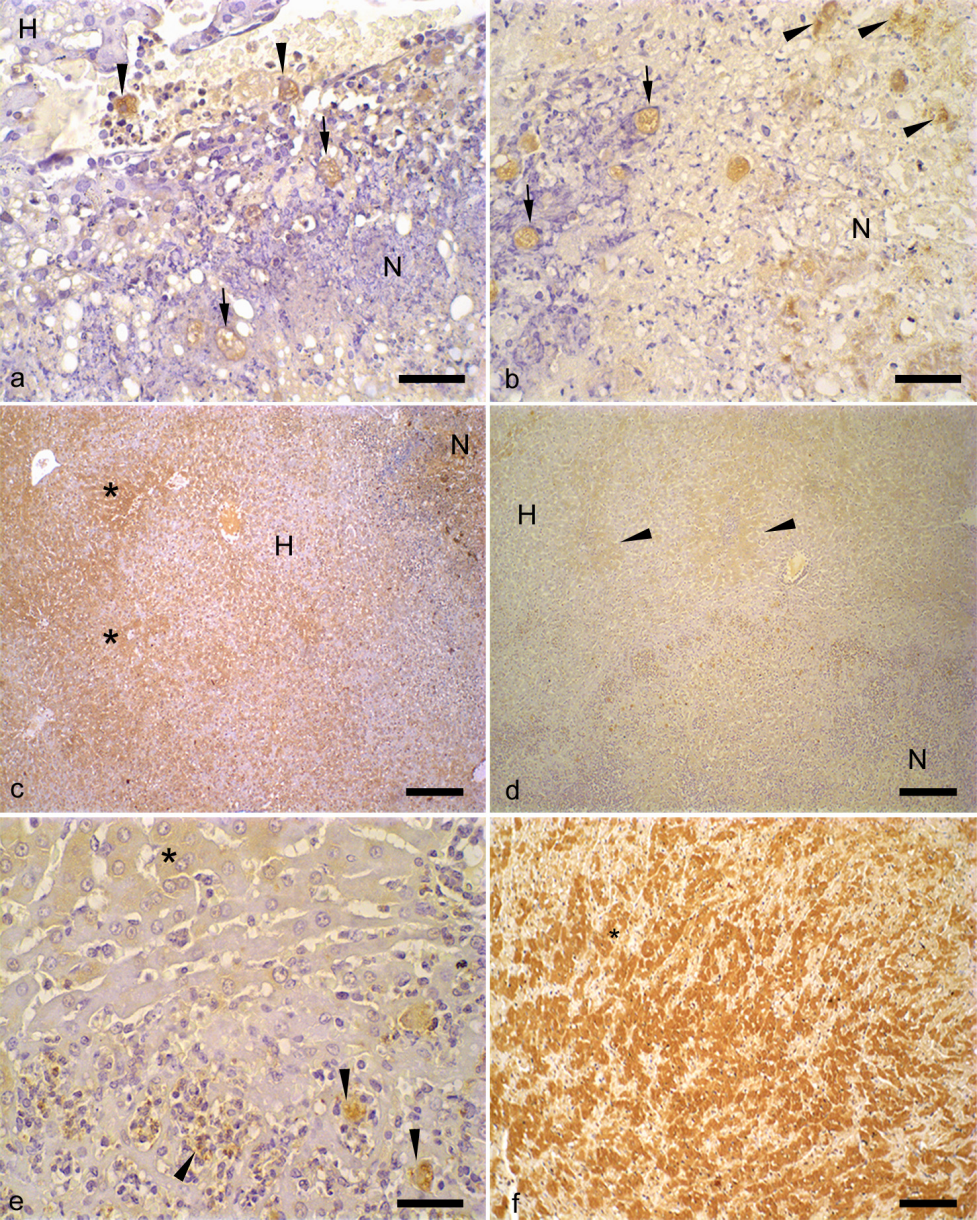
**Immunohistochemical reaction for the components C3 and C9 of the complement in liver of hamsters infected with *E. histolytica *or *E. dispar *trophozoites**. (a) C9^(+) ^trophozoites (arrows) present in the necrotic area (N) induced by *E. histolytica *infection. Note the presence of C9^(+) ^trophozoites inside of the vessels (arrow heads). (H) non-necrotic parenchyma. Bar = 20 μm. (b) Hepatic necrosis (N) produced by *E. dispar*. C9^(+) ^are observed in intact (arrows) and fragmented trophozoites (arrow heads). Bar = 20 μm. (c) Necrotic area produced by *E. histolytica *(N). Extensive areas of positive reaction for the C3 component of the complement are observed (*). (H) non-necrotic parenchyma. Bar = 100 μm. (d) Weak C3^(+) ^hepatic parenchyma of hamsters inoculated with *E. dispar *(arrow heads). (N), necrotic zone. (H) non-necrotic parenchyma. Bar = 100 μm. (e) Amplification of the Figure 4d showing C3^(+) ^hepatocytes (*) and fragmented trophozoites of *E. dispar *(arrow heads). Bar = 20 μm. (f) Positive control for C3 immunoreaction (*) using an ischemic/reperfused rat heart. Bar = 100 μm.

C3^(+) ^and C9^(+) ^trophozoites were detected in the vascular lumen, granulomas and inside and in the border of necrotic areas of *E. dispar*- and *E. histolytica*-infected animals (Figs. 4a and 4b). Also, C3^(-) ^and C9^(-) ^trophozoites were found in these areas. C3^(+) ^and C9^(+) ^trophozoites debris immunostaining was higher in livers of *E. dispar *than in livers of *E. histolytica*, indicating the destruction of these parasites (Figs. 4b and 4e). Diffuse positive immunoreactivity of the C3 component was also observed in normal and injured hepatocytes close or not to nodular lesions and in central areas of necrosis (Figs. 4c and 4d). These findings were much more evident in livers of *E. histolytica *at 24 and 72 h of infection. We used sections from an ischemic/reperfused heart to perform positive controls for C3 and C9. As shown in Figure 4f, large areas of C3 and C9 immunoreactivity were observed in heart sections. No significant staining was detected in the negative controls from liver and heart (data not shown).

**Figure 5:**
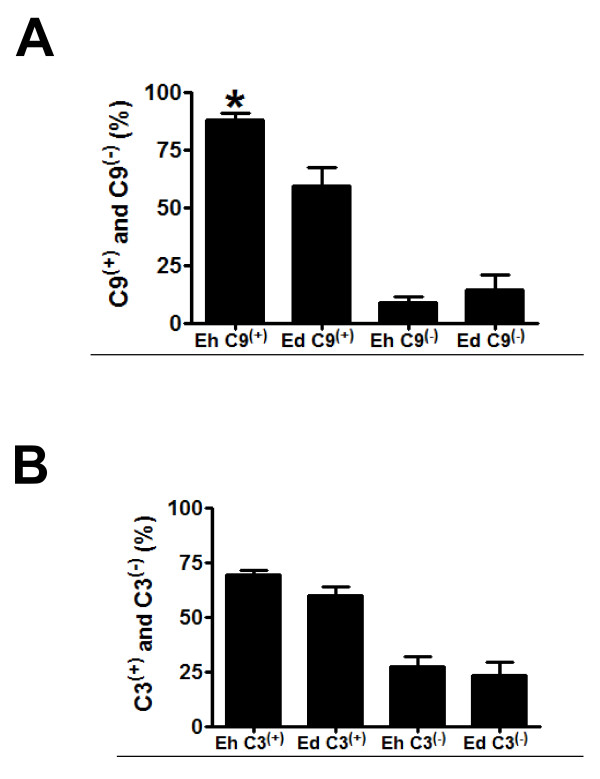
**Quantification of C3 and C9 complement**. (a) Distribution of C9(+) and C9(-) trophozoites and (b) C3(+) and C3(-) trophozoites in liver of hamsters infected with *E. histolytica *or *E. dispar*. Data is expressed as percentage of the positive and negative numbers of trophozoites and shown as means ± SEM. * p < 0.05 vs. *E. dispar *C9(+)trophozoites.

The binding of C9 to *E. histolytica *trophozoites reduced over the period analyzed (12, 24, 48, 72, 144 and 192 h). Figure 5 shows C3^(+)^, C9^(+)^, C3^(-) ^and C9^(-) ^trophozoites distribution in *E. dispar*- and *E. histolytica*-infected animals. Hamsters infected with *E. histolytica *presented a significantly higher C9^(+) ^immunoreactivity when compared with *E. dispar*-infected hamsters. In contrast, no significant differences were observed in the binding of C3 complement to trophozoites over the period analyzed as well as between the strains in each time point. A positive correlation between necrotic area and number of C9^(+) ^trophozoites was observed in animals inoculated with *E. dispar *(p < 0.05).

## Discussion

The most important observation of our present study is the demonstration and quantification *in vivo *of the binding of antibodies against *E. histolytica *and *E. dispar *trophozoites to livers of an experimental model of amoebiasis in hamsters. Moreover, we found that this binding was higher in trophozoites of the non-pathogenic strain *E. dispar *and a positive correlation between binding of the C9 complement to *E. dispar *trophozoites and necrotic area.

The innate immune response is the first reaction of the host against a pathogen and it serves to limit the progression of the infection during the beginning of the exposure of the host to microorganisms. Innate immune response involves the participation of neutrophils, macrophages, natural antibodies and the complement system. The complement system initiates a local inflammatory response which attracts leucocytes to the side of infection and promotes opsonization, leading to removal of pathogens [17]. Such response was observed in our experimental model since it was observed presence of binding of antibodies, C3 and C9 components to trophozoites during all time points of infection. In contrast, Campos-Rodrigues *et al*. [18] only reported significant binding of antibodies to trophozoites up to 72 h after infection. Probably, the antibody binding to trophozoites during the period of 12 to 72 h are natural, while after this interval (144 and 192 h), there are specific antibodies produced by the adaptative immune response. In our study, these antibodies were viewed in trophozoites of both strains; however the antibody binding is significantly higher in *E. dispar *than in *E. histolytica *trophozoites. This finding might be attributed to the lower production of cysteine proteinases, enzymes able to cleave immunoglobulins, by the non-pathogenic specie *E. dispar *[3]. These observations reveal differences in escaping of the immune responses between these two amoebas species in the experimental hepatic abscess.

It has been suggested that the humoral response is not efficient against amoebas *in vivo *[18]. However, occasionally we observed antibodies binding to *E. histolytica *and *E. dispar *trophozoites debris with or without a contact to inflammatory cells. Similarly, C9^(+) ^debris areas were found in infected livers, suggesting *E. histolytica *and *E. dispar *trophozoites destruction. These observations were more evident in *E. dispar*-infected hamsters, probably due to the lower expression of galactose/*N*-acetyl-D-galactosamine inhibitable lectin (CD59-like molecule). In *E. histolytica*, this molecule is related to protection of the trophozoites against the complement action [19]. Thus, because a positive correlation between the amount of C9^(+) ^trophozoites and necrotic area was observed only in animals inoculated with *E. dispar*, these data suggest that C9 deposition on *E. dispar *trophozoites causes their destruction, release of toxic substances and amplification of the necrosis zone. In fact, the amount of trophozoites debris was higher in *E. dispar*-inoculated animals. Consequently, these observations demonstrate the ability of the *E. histolytica *trophozoites in escaping of the complement effects *in vivo *during the hepatic infection.

With regard to the C3 results observed in our study, the larger area of hepatic parenchyma with positive immunoreactivity for this complement in animals inoculated with *E. histolytica *could be due to the ability of this species to produce larger amount of cysteine proteinases since these enzymes are able to activate the complement system [13]. Taking into account that *E. histolytica *is more efficient in escaping the complement actions, the reason by which this amoeba induces a strong and persistent activation of the complement system might be related to the capacity of this strain to manipulate the immunological system in its favor.

In accordance with previous studies, it was observed a small amount of trophozoites in livers at 12 h of infection [11]. At this time point, most of the inflammatory foci did not contain parasites and the sinusoidal capillaries were dilated and with large number of leucocytes, especially neutrophils. It is believed that the survival trophozoites from the initial attempts of the immunological system to heal the infection are responsible for the evolution of the hepatic abscess. In line with this, it is possible that amoeba strains are composed of mixed populations of trophozoites constituted by resistant and sensible parasites [20]. Interestingly, Costa *et al*. reported that the number of trophozoites increased in livers of hamsters infected with both strains at 24 h of inoculation, suggesting that the complement and antibodies actions were not able to completely eliminate the trophozoites [7]. As a consequence, this fact could select subpopulations of resistant parasites and contribute to the development of hepatic abscesses.

## Conclusions

The morphological and immunohistochemical results seen in this work suggest that complement and antibodies were able to destroy trophozoites of both amoebic strains in the liver of experimentally infected hamsters. In this first comparative study, was also demonstrated *in situ *a higher resistance of *E. histolytica *trophozoites to the antibodies response and complement system than *E. dispar*. Although it has been demonstrated that the complement system is not enough to impair the development and progression of the hepatic lesions [18], our current data show that the complement system and antibodies might be able to partially contain the increase of *E. histolytica *trophozoites *in vivo*. On the other hand, in addition other immunological mechanisms might contribute to the pathogenesis of hepatic abscess by selecting resistant trophozoites.

## Competing interests

The authors declare that they have no competing interests.

## Authors' contributions

CCAX, GMA and CMV conceived the study. CCAX and GMA by rearing the amoebas, made the inocula and liver inoculation of hamsters. FAJ made the experiment of the Myocardial Ischemia/Reperfusion. CCAX and CMV made the necropsy of hamsters, histopathologic procedures and morphometry analysis.

CCAX, FAJ and NAC made the immunohistochemical assays. All authors participated in the study design, analysis of the results, drafted the manuscript and have given final approval of the version to be published.
